# The effect of *Allium sativum* in experimental peritoneal adhesion model in rats^1^

**DOI:** 10.1590/s0102-865020190100000002

**Published:** 2019-12-09

**Authors:** Uğur Topal, Nuri Emrah Göret, Ceren Canbey Göret, Ömer Faruk Özkan

**Affiliations:** I MD, Department of General Surgery, Erciyes University Medical Faculty, Melikgazi, Kayseri, Turkey. Conception and design of the study, statistics analysis, manuscript preparation and writing, final approval.; II MD, Department of General Surgery, Health Sciences University, Kartal Dr Lütfi Kırdar Research and Education Hospital, Istanbul, Turkey. Acquisition of data, technical procedures, final approval.; III MD, Department of Surgical Pathology, Health Sciences University, Sancaktepe Research and Education Hospital, Istanbul, Turkey. Conception and design of the study, analysis and interpretation of data, technical procedures, histopathological examinations, manuscript writing, final approval.; IV MD, Department of General Surgery, Health Sciences University, Umraniye Research and Education Hospital, Istanbul, Turkey. Acquisition, analysis and interpretation of data, technical procedures, statistics analysis, manuscript writing, critical revision, final approval.

**Keywords:** Garlic, Lymphadenopathy, Free Radicals, Rats

## Abstract

**Purpose:**

To evaluate the effect of garlic on formation of postoperative adhesions in rats.

**Methods:**

Twenty-four Sprague dawley rats were divided into three groups. In Group 1 (sham), laparotomy was performed and stitched up. In Group 2 (control), after laparotomy was performed, punctate hemorrhage was induced by cecal abrasion in the cecum and 2 cc of saline was intraperitoneally administered to each rat. In Group 3 (experimental), after laparotomy was performed, punctate hemorrhage was induced by cecal abrasion in the cecum and each rat was intraperitoneally administered a sterile Allium sativum derivative. The rats in all groups were re-laparotomized on postoperative day 7; samples were obtained from the peritoneal tissue surrounding the cecum

**Results:**

In Group 3, there was a statistically significant difference in terms of inflammation, lymph node size, and free oxygen radicals; these parameters tended to increase. In terms of fibrosis evaluated using H&E and MT, there was no significant difference between groups 2 and 3.

**Conclusions:**

No positive outcomes indicating that Allium sativum reduces intra-abdominal adhesions were obtained. However, it caused severe inflammation in the tissue. Additionally, in immunohistochemical analyses conducted to detect oxidative stress, allium sativum increased the production of free oxygen radicals in the tissue.

## Introduction

Postoperative peritoneal adhesions (PA), defined as fibrous band formation between intra-abdominal organs, is one of the most important problems of surgery. It is stated that the frequency of PA increases up to 90% in some sources^1^. Abdominal reoperation is more difficult and riskier when there are adhesions. Increased enterotomy risk, longer dissection times and longer operation times are serious consequences of intraabdominal adhesions^2^ ^,^ ^3^.

PAs are caused by the normal peritoneal healing not being limited, due to an increase in vascular permeability as a result of peritoneal damage and release of fibrin-rich exudate. Various drugs, medical agents and surgical techniques have been tried to prevent PPA formation, but there is currently no active and accepted treatment modality. Experimental animal studies are preferred for the studies on prevention of PPAs, mainly because it is difficult and risky to apply to humans, and most of these studies are performed on rats^4^ ^–^ ^7^.

Inflammation and infection secondary to intraabdominal operations may cause adhesions and may be responsible for the etiopathogenesis of lymphadenopathy secondary to inflammation^7^ ^,^ ^8^. In animal models, many products have shown to reduce postoperative adhesion formation, such as corticosteroids, non-steroidal anti-inflammatory drugs, reactive oxygen species (ROS) scavengers, fibrinolytic agents, flotation agents and semisolid barriers and mechanical barriers^8^ ^–^ ^11^.

Allium sativum has been used as a medicine in traditional medicine for centuries and is a well-known substance with anti-inflammatory, antibacterial, fibrinolytic and wound healing properties^8^ ^,^ ^12^. Considering that if allium sativum has an effect on preventing peritoneal adhesion, it can be used in routine practice because it can be found cheap and easy to prevent postoperative adhesion, in addition to examining its effectiveness in preventing intraabdominal adhesions, we aimed to investigate the effects of free oxygen radicals immunohistochemically and the number and size of reactive lymph nodes.

## Methods

### Experiment protocol

This study was performed in the laboratory of Hamidiye Turkey, with the approval of the university's Laboratory Animals Ethics Committee (No: 15.05.2019/2019-05/07).

Rats were obtained from SBU experimental animals center and kept in special cages under appropriate feeding conditions during the study. The rats were housed in special metal cages with controlled heat (between 19-22C) and illumination (08-20 light, 20-08 dark). Rats had normal water and standard foods access without restriction. Prior to the test procedure, all rats were weighed with an analytical balance and their body weights (BW) recorded. Sprague dawley rats weighing 250-300g were divided into three groups (n=8, in each group).

All rats were anesthetized with intramuscular (IM) ketamine (Ketalar 500 mg, 35 mg/kg body weight [BW]; Pfizer) and xylazine (Kepro Xylazine 20, 15 mg/kg BW; Biopharm Veterinary Drugs). After anesthesia, they were cleaned with 10% povidone-iodine solution. A 3 cm median laparotomy was performed. After the procedure, the incision was closed with continuous 3-0 polypropylene sutures (Prolene; Bicakcilar Co., Istanbul, Turkey).

Group 1 (sham): After laparotomy, the abdomen of the rats was closed again without performing any procedure. On postoperative day 7, rats were relaparotomized under anesthesia; peritoneal tissue surrounding the cecum was sampled and rats were sacrificed.

**Figure 1:**
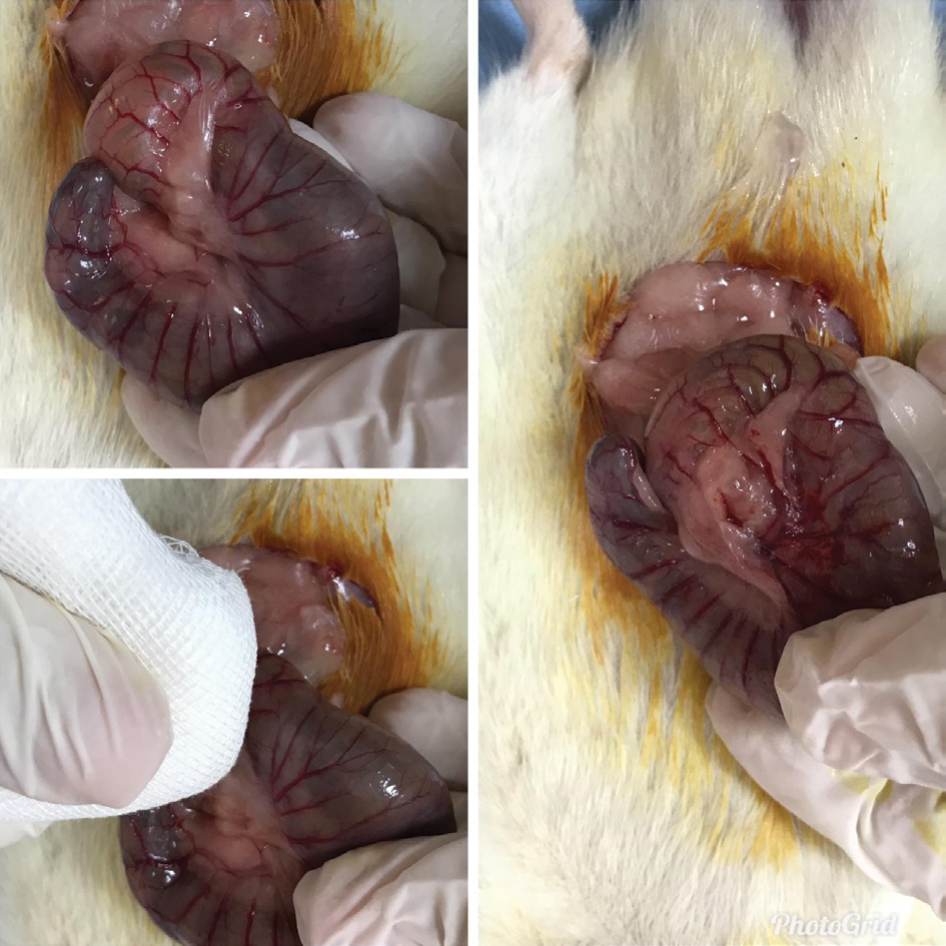
Creation of punctate hemorrhage in the cecum by cecal abrasion after laparotomy.

Group 2 (control group: physiological saline solution): After laparotomy in rats, abrasion, punctate hemorrhage with sterile gauze was created in cecal fat tissue and intraperitoneal 2 cc saline solution was applied. On postoperative day 7, rats were relaparotomized under anesthesia; peritoneal tissue surrounding the cecum was sampled and rats were sacrificed (Fig. 1).

Group 3 (experiment group: allium sativum): After laparotomy in rats, abrasion, punctate hemorrhage with sterile gauze was created in cecal fat tissue and intraperitoneal 5 ml/kg (Oleum Allii SativiR; Arifoglu, Firuzkoy Esenyurt Road No. 32 Avcilar, Istanbul) derivative was applied. On postoperative day 7, rats were relaparotomized under anesthesia; peritoneal tissue surrounding the cecum was sampled and rats were sacrificed.

### Histopathological examination

All materials were fixed with 10% buffered formaldehyde. Samples embedded in paraffin blocks were slit sections of 4-5 micron thickness and stained with hematoxylin & eosin (H&E). In addition to H&E, the samples were sectioned with a thickness of 3-4 microns and stained with Masson's trichrome (MT) histochemically. In addition, 3 micron sections were taken from paraffin blocks and immunohistochemical staining with GSTP1, GLUT-red, SOD1, CAT was performed using LEICA kits in LEICA automatic staining device.

Histopathological effects were evaluated according to the parameters listed below (Table 1):

**Table 1:** Histopathological scoring table.

Score	Fibrosis	Inflammation	Masson Tricrom	SOD1, CAT, GSTP1, GLUT RED (Immunohistochemistry)
0	None	None	None	None
1	Mild	Mild	Mild	Mild
2	Significant	Significant	Significant	Significant
3	Intense	Intense	Intense	Intense

– Fibrosis
– Inflammation
– Number of lymph nodes
– Size of largest lymph node (mm)

Fibrosis Scores with H&E were evaluated as:

No effect: 0
Light effect: 1
Moderate effect: 2
Intense effect: 3

Immunohistochemical staining scores for GSTP1, SOD1, CAT and Glut Red were evaluated as:

0: no staining
1: focal weak positivity
2: moderate positivity
3: intense strong positivity

Histochemical fibrosis evaluation scores with Masson Trichrome (MT) were evaluated as:

0: no staining
1: focal weak positivity
2: moderate positivity
3: intense strong positivity

### Statistical analysis

Data were analyzed by SPSS 24 IBM SPSS Statistics for Windows, version 24 (IBM Corp., Armonk, N.Y., USA). The normal distribution of the variables was examined by analytical methods (Kolmogorow Smirnow test) considering visual (histogram and probability graphs) and sample size. Kruskal-Wallis test was used for comparisons between the groups since not all variables had normal distribution. Descriptive statistics are given as arithmetic mean ± standard deviation and median (quarters). One-Way ANOVA test was used for binary comparisons. Differences between the groups and changes in the number of lymph nodes over time and total score variables were analyzed by two-way variance analysis. Bonferroni test was used for differences between the groups. Statistical significance level was accepted as P <0.05.

## Results

From statistical analyses, the results of specimens in Groups 1, 2, and 3 were not normally distributed; therefore, they were compared using the Kruskal-Wallis test.

**Figure 2:**
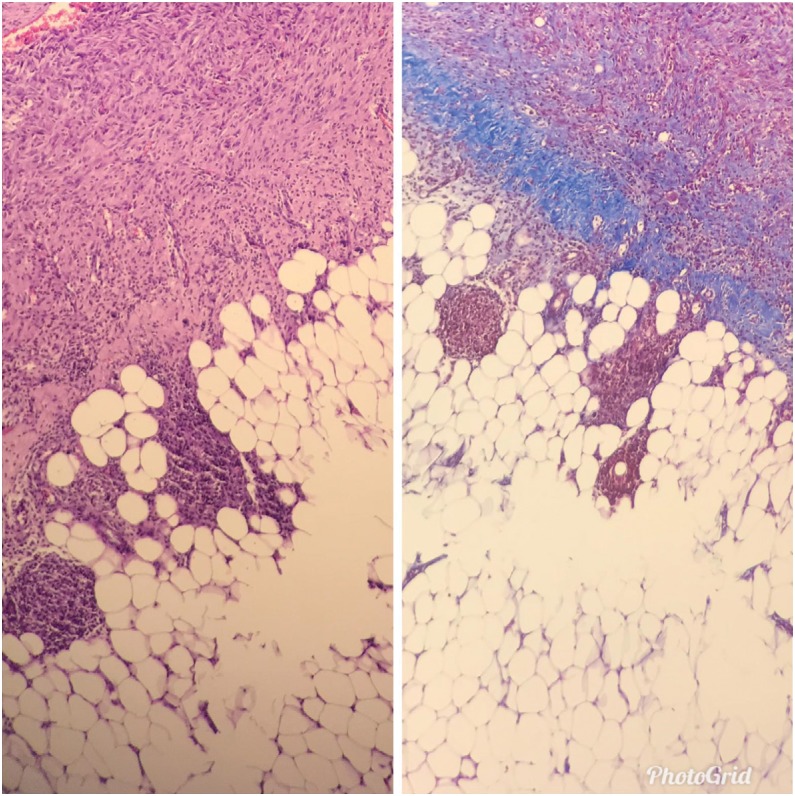
On the left, Score 3 fibrosis, intense (score 2) inflammation with hematoxylin & eosin (H&E x100). On the right, Intense (score 3) fibrosis with Masson's Trichrome (Histochemistry x100).

In Kruskal-Wallis analysis, there was statistical difference between the groups in terms of inflammation, fibrosis, lymph node size and histochemical fibrosis. (Fig. 2). They were higher in Group 3 compared to other groups (p<0.05) (Table 2).

**Table 2:** Kruskal wallis test analysis.

	Fibrosis			Fibrosis with Masson's trichrome			Inflammation			Lymph node size		
	Mean Rank	x^2^	p*	Mean Rank	x^2^	p*	Mean Rank	x^2^	p*	Mean Rank	x^2^	p*
Group 1	6.50	9.088	**0.011**	6.88	8.979	**0.011**	6.75	9.503	**0.009**	6.50	9.088	**0.011**
Group 2	15.44		14.19			13.69			15.44			
Group 3	15.56		16.44			17.06.			15.56			

In the paired group analysis, there was a statistical significance for fibrosis when Group 1 and Group 2 (p=0.018) and Group 1 and Group 3 (p=0.003) were compared, whereas when Group 2 and Group 3 were compared, it was not statistically significant (p=0.483) (Table 3).

**Table 3:** Histopathological evaluation of the study groups.

Groups	Fibrosis	Fibrosis with Masson's trichrome	Inflammation	Lymph node size
Group 1-2	P 0.018*	P 0.018*	P 0.026*	P 0.010*
Group 1-3	P 0.003*	P 0.003*	P 0.003*	P 0.008*
Group 2-3	P 0.483	P 0.483	P 0.179	P 1.000

* *P* <0.05, pair-group analysis was evaluated via the Mann-Whitney U test.

** Group 1: sham group, group 2: control group, group 3: experimental group.

*** Lymph node size was microscopically measured in the histopathological examination in mm.

For fibrosis with Masson's Trichrome, there was a statistical significance when Group 1 and Group 2 (p=0.018) and Group 1 and Group 3 (p=0.003) were compared, whereas when Group 2 and Group 3 were compared, it was not statistically significant (p=0.483).

For inflammation, there was a statistical significance when Group 1 and Group 2 (p=0.026) and Group 1 and Group 3 (p=0.003) were compared, whereas when Group 2 and Group 3 were compared, it was not statistically significant (p=0.179).

For lymph node size, there was a statistical significance when Group 1 and Group 2 (p=0.01) and Group 1 and Group 3 (p=0.007) were compared, whereas when Group 2 and Group 3 were compared, it was not statistically significant (p=1.000).

The total immunohistochemical score and number of lymph nodes were normally distributed, and statistical evaluation between the three groups was done using one-way ANOVA.

When the groups were compared in terms of immunohistochemical score, the mean values were 1.75 in Group 1, 5.25 in Group 2, and 4.33 in Group 3. The P-value was 0.0001 (P < 0.005), and there was a significant difference (Table 4).

**Table 4:** Results of comparison with immunohistochemical score groups.

	Groups			F	p
	Group A	Group B	Group C		
Immunohistochemical score	1.75+2.37 0.0-7.0	5.25+1.03 4.0-7.0	6.0+2.07 4.0-9.0	11.227	**0.000**

In the paired group analysis in terms of immunohistochemical score, there was a statistical significance between Group 1 and Group 2 (p=0.004) and Group 1 and Group 3 (p=0.001), whereas the difference between Group 2 and Group 3 was not statistically significant (p=1.000) (Table 5).

**Table 5:** Comparison between the groups with respect to the immunohistochemical score and the number of lymph nodes.

Groups	Number of lymph nodes	Immunohistochemical score
Group 1-2	1.000	0.004*
Group 1-3	1.000	0.001*
Group 2-3	1.000	1.000

* The mean difference is significant at the level of 0.15.

** Post hoc pair-group analysis was performed using Bonferroni correction.

*** Statistical evaluation between the three groups was assessed with one-way ANOVA.

**** Each immunohistochemical marker was individually scored, but statistical evaluation was performed for each rat on the basis of the total score (SOD-1, CAT, GSTP-1, Glut Red).

***** In the histopathological examination, the number of lymph nodes was microscopically counted for each rat.

Each immunohistochemical marker was individually scored, but statistical evaluation was performed for each rat on the basis of the total score (SOD-1, CAT, GSTP-1, Glut Red). Statistical evaluation between the three groups was done using one-way ANOVA.

**Figure 3:**
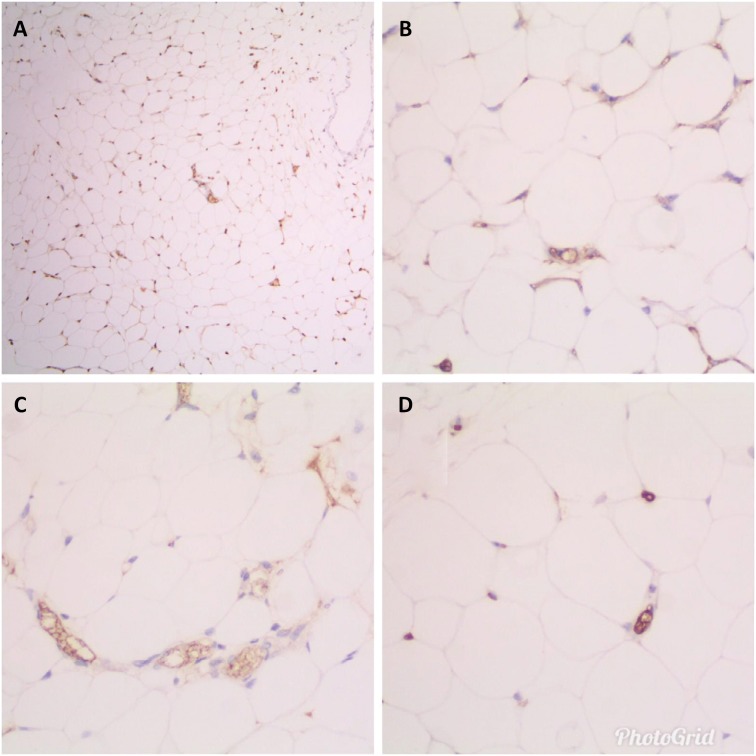
A. Intense (Score 3) staining of adipose tissue with SOD 1 (IHC x40). **B**. Moderate positivity (Score 2) with GSTP 1 (IHC x400). **C**. Moderate positive (Score 2) staining with CAT (IHC x400). **D**. Light (Score 1) positive staining with GLUT RED (IHC x400).

In immunohistochemical analyses (SOD-1, Glut Red, CAT, GSTP-1) on free oxygen radicals, allium sativum statistically increased the production of free oxygen radicals in the tissues (Fig. 3).

In terms of the number of lymph nodes, the mean values were 3.1111 in Group 1, 3.6667 in Group 2, and 1.4444 in Group 3. P value was 0.934 (P> 0.005) and there was no statistically significant difference (Table 6).

**Table 6:** Mean values of free oxygen radicals number of lymph nodes.

Groups	Number of lymph nodes	Free oxygen radicals with immunohistochemical
Group 1	Mean: 3.63	Mean: 1.7500
	St. deviation: 1.923	St. deviation: 2.37547
	Median: 4	Median: 1.00

Group 2	Mean: 3.75	Mean: 5.2500
	St. deviation: 1.753	St. deviation: 1.03510
	Median: 4	Median: 5

Group 3	Mean: 4.00	Mean: 4.3333
	St. deviation: 2.449	St. deviation: 2.63202
	Median: 3.50	Median: 4.50

* The mean difference is significant at the level of 0.15.

** Post hoc pair-group analysis was performed using Bonferroni correction.

*** Statistical evaluation between the three groups was assessed with one-way ANOVA.

**** Each immunohistochemical marker was individually scored, but statistical evaluation was performed for each rat on the basis of the total score (SOD-1, CAT, GSTP-1, Glut Red).

***** In the histopathological examination, the number of lymph nodes was microscopically counted for each rat.

In the paired group analysis for the number of lymph nodes, the P value was 1.0 between Groups 1 and 2, 1.0 between Groups 1 and 3, and 1.0 between Groups 2 and 3 (Table 5).

Post-hoc paired group analyses were evaluated using Bonferroni correction *** for total immunohistochemical score and number of lymph nodes (Mean difference was significant at 0.15.).

## Discussion

Peritoneal adhesions (PA) remain a major health problem and, according to some sources, have been reported to be seen at very high rates, especially after abdominal surgery^1^. Intestinal obstruction, abdominal pain due to visceral peritoneal traction, infertility due to extraluminal compression, urinary dysfunction due to traction of ureters may be seen secondary to PA^13^.

PA may be congenital or especially acquired in the postoperative period^13^. In a postmortem study, approximately 1/3 of the cases with peritoneal adhesion were found to be non-surgical cases and endometriosis, peritoneal dialysis and radiotherapy were among the events that triggered intraabdominal inflammation, but the majority of cases were reported in the postoperative period^14^.

In case of a possible re-operation in PA cases, prolongation of operation and dissection times and increased risk of surgical complications can be mentioned^2^ ^,^ ^3^.

Multiple strategies have been proposed to eliminate PA, but there are varying results^15^.

After any intervention in the peritoneal tissue (surgery, inflammation of the trauma, infection or placement of foreign bodies in the peritoneal cavity, etc.), the development of adhesion may begin.

After injury/damage to the mesothelial cells covering the peritoneal surface, the healing process begins. Vasoactive amines such as histamine and quinine contribute to the accumulation of fibrin-rich exudates around the damaged area by increasing vascular permeability.

As the fibrin polymers in the exudate interact with the fibronectin to form the fibrin gel matrix, which produces fibrin bands between the injured area, simultaneous fibrinolytic activity begins. Fibrinolytic activity is more dominant in areas where healing occurs without adhesion. In contrast, if there is a defect or imbalance in fibrinolytic activity, there is persistence of fibrinous material in these areas. Secondary to this, proliferative fibroblasts migrate to this area and accumulate extracellular matrix material that contains collagen that contributes to adhesion formation. Thus, the different mechanical steps, some of which are described above, regulate the healing process and the presence of imbalances in any of them potentially contributes to the development of adhesion^7^ ^,^ ^16^.

Free oxygen radicals are molecules that are released from polymorphic leukocytes and macrophages and show their effects in the early stage of inflammation. Free radicals target DNA, proteins and lipids, attack membrane lipids and cause peroxidation. This causes damage to the cell membranes and in this case increases the formation of microvascular edema. In recent years, it has been shown that oxygen-derived free radicals and metabolites play a role in increased cell and tissue damage in leukocyte-dependent inflammatory reactions and that these radicals cause secondary damage in many tissues^17^ ^,^ ^18^. As a result, inflammation and fibrosis are observed histopathologically on a microscopic level.

Adhesions have shown to be a result of incorrect healing caused by injured peritoneal tissues associated with oxidative stress. In addition to parameters such as fibrosis and inflammation, GSTP-1, GLUT Red, SOD-1 and CAT were used to determine oxidative stress parameters immunohistochemically in tissues. On postoperative day 7, it was reported that these adhesive strips progressed to permanent fibrous adhesions, and that hypoxia directly promoted the production of free oxygen radicals in tissues^7^ ^,^ ^8^.

Various antioxidant agents have been used to prevent adhesion formation by preventing tissue damage caused by free oxygen radicals. Nitric oxide (NO) can prevent adhesion by decreasing free oxygen radicals due to its superoxide binding properties. Phosphodiesterase inhibitors such as sildenafil citrate increasing NO levels have experimentally shown to inhibit adhesion^19^.

Anti-inflammatory properties of Allium sativum have been previously reported. Allium sativum has been reported to inhibit anti-inflammatory activity by inhibiting activation of nuclear factor kappa-B (NF-kB) caused by oxidative stress^20^. In addition, it is reported in the literature that allium sativum inhibits the production of T helper 1 cells and inflammatory cytokines, increasing corticosteroid levels, and acts as an anti-inflammatory and immunomodulating agent^21^.

Rassoul *et al*.^22^, and Kuo *et al*.^23^ showed that Allium sativum extract showed an anti-inflammatory effect by inhibiting the expression of IL-1 alpha-derived vascular cell adhesion molecule-1 (VCAM-1) and intracellular adhesion molecule-1 (ICAM-1).

In a study by Şahbaz *et al*.^8^ Allium sativum was shown to be effective in preventing peritoneal adhesion on postoperative day 10 due to its fibrinolytic, antithrombotic, anti-inflammatory and antioxidant effects.

In addition to inflammation and fibrosis, many reactive lymphadenopathies of various sizes were detected in almost all groups. Reactive lymphadenopathy usually occurs due to infections, inflammation or malignancy. Lymph nodes have functions such as microorganism filtration and antibody production. Lymph nodes grow in the presence of microorganisms, malignant cells, or antigenic reactions, which cause the proliferation of lymphocytes or macrophage hyperplasia^24^.

In a study similar to ours, Goret *et al*.^7^ found in their 2018 study that picnogenol-induced foreign body reaction and lymph node number increased in peritoneal adhesion model. In their study, they reported that the presence of acute inflammation, which later became chronic, played a role in the etiology of lymphoid hyperplasia, which was statistically significant, especially in the group receiving picnogenol.

However, contrary to the results in the literature, we could not detect any positive effects of Allium sativum in addition to daily routine procedures on inflammation, fibrosis or lymphoid hyperplasia. In addition, no benefit of allium sativum was detected in immunohistochemical analysis to detect oxidative stress.

Although the positive effect of Allium sativum on peritoneal adhesion has been reported in the literature, with the early period findings in our study, it was found that this substance did not provide an additional contribution to the current protocol.

Various studies are needed to test and investigate the effects of this type of traditional medicine used in PA prevention.

## Conclusion

In this study, no positive results were found that Allium sativum had positive effects on fibrosis, inflammation or reactive lymph node size/number which are among the intraabdominal adhesion parameters; in addition, immunohistochemical analysis to detect oxidative stress at the cellular level did not show a positive decrease in free oxygen radicals.
